# Ventricular Tachycardia Ablation Outcomes in Patients Without Previous Implantable Cardioverter-Defibrillator Insertion: A Propensity Score–matched Analysis

**DOI:** 10.19102/icrm.2026.17033

**Published:** 2026-03-15

**Authors:** Ali Saad Al-Shammari, Ankur Singla, Ameer Awashra, Adishwar Rao, Ahmed Sermed Al Sakini, Ahmed Al-Kaisey, Tarique Ahmed, Sima Rawal, Mohammed Saifuddin, Mohamed Wagdy, Muhammad Bilal Munir, Saif Syed, Keerat Rai Ahuja, Yasar Sattar

**Affiliations:** 1 Department of Internal Medicine, College of Medicine, University of Baghdad, Baghdad, Iraq; 2 Department of Internal Medicine, Northwest Health–Porter, Valparaiso, IN, USA; 3 Department of Internal Medicine, An Najah National University, Nablus, Palestine; 4 Department of Internal Medicine, Guthrie Robert Packer Hospital, Sayre, PA, USA; 5 Department of Cardiology, Royal Melbourne Hospital, Melbourne, Australia; 6 Department of Cardiology, Doctor Soliman Fakeeh Hospital, Jeddah, Kingdom of Saudi Arabia; 7 Department of Internal Medicine, Arnot Ogden Medical Center, Elmira, NY, USA; 8 Department of Internal Medicine, Navodaya Medical College, Raichur, India; 9 Department of Internal Medicine, Modern University for Technology and Information, Cairo, Egypt; 10 Department of Cardiology, University of California, Davis, Sacramento, CA, USA; 11 Department of Internal Medicine, Royal College of Surgeons in Ireland, Dublin, Ireland; 12 Department of Cardiology, West Virginia University Camden Clark Medical Center, Parkersburg, WV, USA

**Keywords:** Catheter ablation, propensity score matching, ventricular tachycardia

## Abstract

Ventricular tachycardia (VT) is a fatal arrhythmia, often managed with implantable cardioverter-defibrillators (ICDs). Many patients, however, present without an ICD. The role of catheter ablation in this high-risk group is unclear, particularly for short-term in-hospital outcomes. We assessed associations between ablation and in-hospital outcomes among ICD-naive VT patients using a large national dataset. We conducted a retrospective study using the National Inpatient Sample (2016–2021), identifying adult hospitalizations with VT. Patients with prior ICDs or ICD implantation during the same admission were excluded. The cohort was divided into those who underwent catheter ablation versus those managed without ablation. Multivariable logistic regression and 1:1 propensity score matching (PSM) adjusted for demographic, clinical, and hospital factors. The primary outcome was in-hospital mortality; secondary outcomes included ST-elevation myocardial infarction (STEMI), sepsis, major adverse cardiac events (MACEs) (death, STEMI, or cardiogenic shock), cardiogenic shock, tamponade, mechanical circulatory support (MCS), acute heart failure, and prolonged hospitalization (≥7 days). Of 2,214,424 VT hospitalizations, 32,640 (1.5%) underwent catheter ablation. After PSM (n = 12,668), ablation was associated with significantly lower rates of in-hospital mortality (3.17% vs. 8.98%; *P* < .001), STEMI (6.82% vs. 18.83%; *P* < .001), sepsis (3.38% vs. 10.34%; *P* < .001), and MACEs (15.82% vs. 28.40%; *P* < .001). However, ablation was associated with higher rates of cardiac tamponade (1.78% vs. 0.43%; *P* < .001), cardiogenic shock (9.14% vs. 7.12%; *P* < .001), and MCS use (5.04% vs. 3.71%; *P* < .001). Rates of acute heart failure and prolonged hospitalization were comparable. In ICD-naive VT patients, catheter ablation was associated with improved in-hospital survival and fewer complications, albeit with higher procedural risks.

## Introduction

Ventricular tachycardia (VT) is a life-threatening arrhythmia often manifested in patients with structural heart diseases. A notable subset of patients hospitalized with VT do not have an implantable cardioverter-defibrillator (ICD) at presentation because of clinical ineligibility, postponed evaluation, or systemic disparities, despite ICDs being the gold standard for preventing sudden cardiac death.^1^ Catheter ablation has emerged as an integral treatment for VT, particularly in those with ICDs. Landmark trials such as VTACH (“Substrate Modification in Stable Ventricular Tachycardia in Addition to Implantable Cardioverter Defibrillator Therapy”) and VANISH (“Ventricular Tachycardia Ablation or Escalated Drug Therapy”) demonstrated that ablation reduces VT recurrence and ICD therapies in ischemic cardiomyopathy in ICD recipients.^2,3^ However, there are limited data within published literature on outcomes in ICD-naive patients. In a multicenter retrospective cohort of patients with genetic channelopathies such as arrhythmogenic right ventricular cardiomyopathy undergoing catheter ablation without prior ICD, Santangeli et al.^4^ reported 72% with acute success and no arrhythmic deaths over a median follow-up of 52 months, although VT recurrences occurred in 19% of the patients. Another recent cohort study assessed VT ablation in ischemic cardiomyopathy patients without an ICD. In this study of 114 individuals, VT recurred in 39.5% over 54 months, but only 5.3% died. Notably, epicardial ablation correlated with lower recurrence rates (8.3% vs. 43.1%).^5^ These previously published findings suggest that catheter ablation could be safe and effective in selected ICD-naive patients. However, prior studies have been limited by small cohorts, methodological limitations, and the lack of non-ablation control arms. Importantly, these analyses focus on long-term arrhythmia outcomes and do not address the impact on short-term in-hospital endpoints (mortality, sepsis, myocardial infarction [MI], major adverse cardiac events [MACEs]). Despite advancements in catheter ablation techniques, the clinical utility of this intervention in VT patients without prior ICD therapy remains insufficiently defined. In particular, there is a lack of large-scale evidence evaluating whether early ablation confers meaningful short-term clinical benefits during the index hospitalization in this high-risk, ICD-naive subgroup. Clarifying these associations is critical, as it could guide early procedural decisions and improve acute care strategies. To address this unmet need, we conducted a national comparative analysis using real-world inpatient data to examine the association of catheter ablation in this population.

## Methods

### Data source

**Figure 1:**
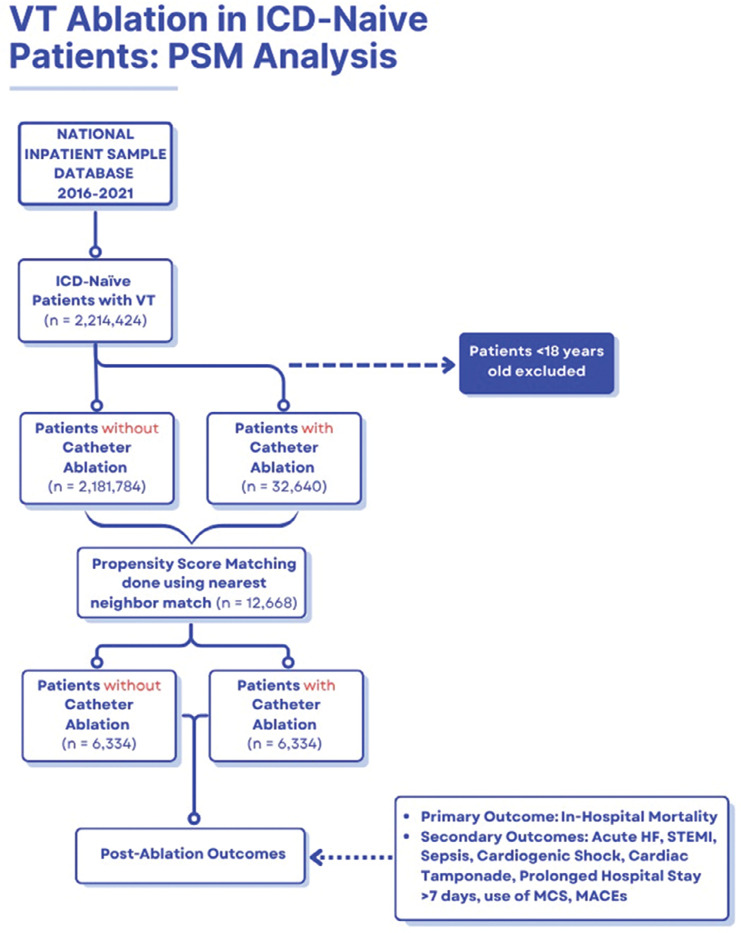
Flowchart of the study. *Abbreviations:* HF, heart failure; ICD, implantable cardioverter-defibrillator; PSM, propensity score matching; STEMI, ST-elevation myocardial infarction; VT, ventricular tachycardia; MACE, major adverse cardiac events; MCS, mechanical circulatory support.

We performed a retrospective observational cohort study using the National Inpatient Sample (NIS), a stratified 20% sample of discharges from United States (US) community hospitals maintained by the Healthcare Cost and Utilization Project (HCUP). The NIS supports weighted, nationally representative estimates. The study period was January 1, 2016, to December 31, 2021 **(Figure 1)**. All analyses complied with the HCUP Data Use Agreement. Because this study used de-identified, publicly available NIS data, institutional review board approval and informed consent were not required in accordance with HCUP policies (available at https://www.hcup-us.ahrq.gov/nisoverview.jsp).

### Study population and exposure

We included adults ≥18 years with VT identified using validated International Classification of Diseases, Tenth Revision (ICD-10) codes. Exposure was catheter ablation during the index hospitalization, identified by ICD-10 Procedure Coding System (PCS) codes. To ensure an ICD-naive cohort, we excluded patients with prior ICDs and those undergoing ICD implantation, revision, or removal during admission. This approach aimed to isolate the association of ablation on short-term outcomes, as ICDs mainly prevent long-term sudden death. The NIS does not reliably distinguish ischemic, nonischemic, or idiopathic VT; therefore, all VT admissions were included, with proxies such as prior MI, cardiomyopathies, and revascularization procedures captured as covariates. Admissions with premature ventricular complexes alone were excluded. Current procedural terminology (CPT) codes were not used. Full inclusion, exposure, and exclusion codes are provided in **Supplementary Table S1**.

**Table S1:** Cohort Definition and Exclusions

Variable	Role	Code(s)/Pattern	Code Set	Position(s)	Notes
Ventricular tachycardia (index)	Cohort inclusion	I47.2	ICD-10-CM	Any diagnosis	Identifies VT admissions
Catheter ablation	Exposure	02583ZZ	ICD-10-PCS	Any procedure	Destruction of cardiac conduction mechanism (percutaneous)
ICD present (history)	Cohort exclusion	Z95.810	ICD-10-CM	Any diagnosis	Exclude to enforce ICD-naive cohort
ICD system placed this admission (generator/S-ICD)	Cohort exclusion	0JH608Z, 0JH638Z, 0JH609Z, 0JH639Z, 0JH60FZ, 0JH63FZ, 0JH808Z, 0JH838Z, 0JH839Z	ICD-10-PCS	Any procedure	Exclude if new ICD/CRT-D or S-ICD placed
ICD lead insertion	Cohort exclusion	02H63KZ, 02HK3KZ, 02HL3KZ, 02H43KZ, 02H73KZ	ICD-10-PCS	Any procedure	Exclude transvenous lead procedures
ICD lead removal/revision	Cohort exclusion	02PA0MZ, 02PA3MZ, 0JPT0FZ, 0JPT3FZ	ICD-10-PCS	Any procedure	Excluded from our study
CPT reference (not used for NIS)	Documentation only	93654	CPT	—	Reported in text; not used for inpatient NIS extraction

*Abbreviations:* CPT, current procedural terminology; CRT-D, cardiac resynchronization therapy defibrillator; ICD, implantable cardioverter-defibrillator; ICD-10-CM, International Classification of Diseases, Tenth Revision, Clinical Modification; ICD-10-PCS, International Classification of Diseases, Tenth Revision, Procedure Coding System; NIS, National Inpatient Sample; S-ICD, subcutaneous implantable cardioverter-defibrillator; VT, ventricular tachycardia.

### Covariates and baseline characteristics

Baseline variables included age; sex; race/ethnicity; primary payer; and hospital region, location/teaching status, and bed size (standard NIS fields). Clinical comorbidities captured for descriptive, matching, and adjustment purposes were as follows: hyperlipidemia, hypertension, chronic heart failure, prior myocardial infarction, prior percutaneous coronary intervention (PCI), prior coronary artery bypass grafting (CABG), obesity, tobacco use, chronic obstructive pulmonary disease (COPD), obstructive sleep apnea (OSA), prior stroke, liver disease, diabetes mellitus, chronic kidney disease/end-stage renal disease (CKD/ESRD), hypothyroidism, nutritional anemia, dilated cardiomyopathy (DCM), hypertrophic cardiomyopathy (HCM), and coronavirus disease 2019 (COVID-19). Comorbidities were identified using ICD-10-Clinical Modification (CM) patterns derived from validated algorithms (Elixhauser family, where applicable). Variable mappings and code patterns are summarized in **Supplementary Tables S2 and S3**.

**Table S2:** Baseline Demographics and Hospital (NIS Fields)

Variable	Role	Code(s)/Pattern	Code Set	Position(s)	Notes
Sex	Baseline/descriptive	—	NIS variable	FEMALE	1 = female, 0 = male
Age group	Baseline/descriptive	—	NIS variable	AGE	18–44, 45–64, and ≥65 years
Race	Baseline/descriptive	—	NIS variable	RACE	White, Black, Hispanic, Asian/Pacific Islander, Native American, Other
Primary insurance	Baseline/descriptive	—	NIS variable	PAY1	Medicare, Medicaid, private, self-pay, no charge, other
Region of hospital	Baseline/descriptive	—	NIS variable	HOSP_REGION	Northeast, Midwest, South, West
Location/teaching	Baseline/descriptive	—	NIS variable	HOSP_LOCTEACH	Rural, urban nonteaching, urban teaching
Bed size	Baseline/descriptive	—	NIS variable	HOSP_BEDSIZE	Small, medium, large

*Abbreviation:* NIS, National Inpatient Sample. “*” = any subcode; ranges use “–” (en dash). Unless specified, ICD-10-CM and ICD-10-PCS codes were queried in ANY diagnosis/procedure position for the index hospitalization. Adults ≥18 years only were considered. CPT codes were NOT used for NIS extraction.

**Table S3:** Baseline Comorbidities (ICD-10-CM; Analysis Use)

Variable	Role	Code(s)/Pattern	Code Set	Position(s)	Notes
Hyperlipidemia	Baseline/PSM and adjust	E78.5	ICD-10-CM	Any dx	Unspecified hyperlipidemia
Hypertension	Baseline/PSM and adjust	I10	ICD-10-CM	Any dx	Essential hypertension
Chronic heart failure	Baseline/PSM and adjust	I50.*	ICD-10-CM	Any dx	Chronic HF at baseline
Prior myocardial infarction	Baseline/PSM and adjust	I25.2	ICD-10-CM	Any dx	Old MI
Prior PCI	Baseline/PSM and adjust	Z98.61	ICD-10-CM	Any dx	Coronary angioplasty implant/graft status
Prior CABG	Baseline/PSM and adjust	Z95.1	ICD-10-CM	Any dx	Aortocoronary bypass graft status
Obesity	Baseline/PSM and adjust	E66.*	ICD-10-CM	Any dx	Includes morbid obesity (E66.01)
Smoker/tobacco use	Baseline/PSM and adjust	F17.2*, Z72.0	ICD-10-CM	Any dx	Nicotine dependency/tobacco use
COPD	Baseline/PSM and adjust	J44.*	ICD-10-CM	Any dx	Chronic obstructive pulmonary disease
Obstructive sleep apnea	Baseline/PSM and adjust	G47.33	ICD-10-CM	Any dx	OSA
Prior stroke	Baseline/PSM and adjust	I69.* or Z86.73	ICD-10-CM	Any dx	Sequelae of cerebrovascular disease or personal history
Liver disease	Baseline/PSM and adjust	Elixhauser (ICD-10-CM set)	ICD-10-CM	Any dx	HCUP Elixhauser definition (software-refined)
Diabetes mellitus	Baseline/PSM and adjust	E10–E14.*	ICD-10-CM	Any dx	Type 1/2 and other diabetes families
CKD/ESRD	Baseline/PSM and adjust	N18.* (± Z99.2)	ICD-10-CM	Any dx	CKD stages; dialysis dependency (Z99.2) if available
Hypothyroidism	Baseline/PSM and adjust	E03.*	ICD-10-CM	Any dx	Hypothyroidism family
Nutritional anemia	Baseline/PSM and adjust	D50–D53.*	ICD-10-CM	Any dx	Iron, B12, folate deficiency
DCM	Baseline/PSM and adjust	I42.0	ICD-10-CM	Any dx	DCM
HCM	Baseline/PSM and adjust	I42.1, I42.2	ICD-10-CM	Any dx	Obstructive/nonobstructive HCM
COVID-19	Baseline/PSM and adjust	U07.1 (2020+)	ICD-10-CM	Any dx	Valid from 2020 onwards

*Abbreviations:* CABG, coronary artery bypass graft; CKD/ESRD, chronic kidney disease/end-stage renal disease; COPD, chronic obstructive pulmonary disease; DCM, dilated cardiomyopathy; HF, heart failure; HCM, hypertrophic cardiomyopathy; ICD-10-CM, International Classification of Diseases, Tenth Revision, Clinical Modification; MI, myocardial infarction; OSA, obstructive sleep apnea; PCI, percutaneous coronary intervention; PSM, propensity score matching. “*” = any subcode; ranges use “–” (en dash). Unless specified, ICD-10-CM and ICD-10-PCS codes were queried in ANY diagnosis/procedure position for the index hospitalization. Adults ≥18 years only were considered. CPT codes were NOT used for NIS extraction.

### Outcomes

The primary outcome was in-hospital mortality. Secondary outcomes were ST-elevation myocardial infarction (STEMI); cardiogenic shock; mechanical circulatory support (MCS) use; sepsis; acute heart failure; cardiac tamponade; length of stay (LOS) ≥7 days; and MACEs defined as a composite of in-hospital death, STEMI, or cardiogenic shock. Outcomes were identified using ICD-10-CM/-PCS code families or derived from standard NIS fields; full operational definitions and code lists appear in **Supplementary Table S4**.

**Table S4:** Definitions and Coding for Outcomes

Outcome	Role	Code(s)/Pattern	Code Set	Position(s)	Logic/Notes
In-hospital mortality	Primary outcome	—	NIS variable	DISPUNIFORM/DIED	Discharge disposition (expired)
STEMI	Secondary outcome	I21.0*, I21.1*, I21.2*, I21.3	ICD-10-CM	Any dx	Exclude I21.4 (NSTEMI)
Acute heart failure	Secondary outcome	I50.21, I50.23, I50.31, I50.33, I50.41, I50.43, I50.811, I50.813	ICD-10-CM	Any dx	Acute/acute-on-chronic only
Cardiogenic shock	Secondary outcome	R57.0	ICD-10-CM	Any dx	
MCS	Secondary outcome	5A02210 (IABP), 5A0221D (Impella^®^ support), 02HA3RZ (Impella^®^ /Tandem insertion), 5A15223 (ECMO)	ICD-10-PCS	Any procedure	Count any of the listed
MACEs (death/STEMI/CS)	Composite outcome	Composite of the above	—	—	Any in-hospital death, STEMI, or cardiogenic shock
Cardiac tamponade	Safety outcome	I31.4	ICD-10-CM	Any dx	Pericardial effusion (tamponade)
Sepsis	Secondary outcome	A40.*, A41.*, R65.20, R65.21	ICD-10-CM	Any dx	Sepsis families including severe sepsis ± shock
LOS ≥7 days	Utilization outcome	—	Derived	LOS	LOS computed from NIS; threshold ≥7 days

*Abbreviations:* CS, cardiogenic shock; ECMO, extracorporeal membrane oxygenation; IABP, intra-aortic balloon pump; ICD-10-CM, International Classification of Diseases, Tenth Revision, Clinical Modification; ICD-10-PCS, International Classification of Diseases, Tenth Revision, Procedure Coding System; LOS, length of stay; MACE, major adverse cardiovascular event; MI, myocardial infarction; NIS, National Inpatient Sample; NSTEMI, non–ST-elevation myocardial infarction; OR, odds ratio; STEMI, ST-elevation myocardial infarction. “*” = any subcode; ranges use “–” (en dash). Unless specified, ICD-10-CM and ICD-10-PCS codes were queried in ANY diagnosis/procedure position for the index hospitalization. Adults ≥18 years only were considered. CPT codes were NOT used for NIS extraction. Impella^®^ is a product of Abiomed (Danvers, MA, USA).

### Statistical analysis

We used methods appropriate for NIS’s complex survey design, applying discharge weights to generate national estimates and accounting for stratification and clustering. Baseline characteristics were compared using design-adjusted chi-squared tests (categorical) and *t* tests (continuous). Associations between ablation and outcomes were estimated using survey-adjusted multivariable logistic regression, reporting adjusted odds ratios (aORs) with 95% confidence intervals (CIs). To mitigate confounding by indication, we performed 1:1 propensity score matching (PSM) using nearest-neighbor matching without replacement. The propensity model included demographics, payer, hospital characteristics, and all comorbidities listed in the outcomes section. Covariate balance was assessed using standardized mean differences (SMDs), with an SMD of <0.10 indicating acceptable balance. Post-match associations were estimated with conditional logistic regression. All analyses were conducted using Stata 17.0 (StataCorp, College Station, TX, USA). Two-sided *P* < .05 was considered statistically significant.

## Results

A total of 2,214,424 patients with a diagnosis of VT without an ICD were identified between 2016 and 2021. Among these, 32,640 (1.5%) underwent catheter ablation, while 2,181,784 (98.5%) did not. Baseline characteristics are shown in **Table 1**. **Table 2** presents unadjusted outcomes, and **Table 3** provides adjusted and propensity score-matched outcomes.

**Table 1:** Baseline Characteristics of the Study

	Ablation	No Ablation	*P* Value
N	32,640 (1.5%)	2,181,784 (98.5%)	
Indicator of sex			
Male	23,515 (72.0%)	1,422,370 (65.2%)	<.001
Female	9125 (28.0%)	759,415 (34.8%)	
Age group (years)			
18–<44	3025 (9.3%)	137,220 (6.3%)	<.001
45–<64	11,480 (35.2%)	645,585 (29.6%)	
≥65	18,135 (55.6%)	1,398,979 (64.1%)	
Race			
White	24,070 (76.0%)	1,501,734 (70.8%)	<.001
Black	3830 (12.1%)	371,470 (17.5%)	
Hispanic	2035 (6.4%)	144,290 (6.8%)	
Asian or Pacific Islander	635 (2.0%)	42,610 (2.0%)	
Native American	140 (0.4%)	10,620 (0.5%)	
Other	960 (3.0%)	51,570 (2.4%)	
Primary insurance			
Medicare	18,485 (56.7%)	1,421,649 (65.2%)	<.001
Medicaid	2680 (8.2%)	222,420 (10.2%)	
Private Insurance	9635 (29.6%)	408,470 (18.7%)	
Self-pay	620 (1.9%)	65,260 (3.0%)	
No charge	95 (0.3%)	5235 (0.2%)	
Other	1070 (3.3%)	56,010 (2.6%)	
Region of hospital			
Northeast	7490 (22.9%)	420,880 (19.3%)	<.001
Midwest	6965 (21.3%)	510,715 (23.4%)	
South	13,125 (40.2%)	851,130 (39.0%)	
West	5060 (15.5%)	399,059 (18.3%)	
Location/teaching status of hospital			
Rural	550 (1.7%)	122,420 (5.6%)	<.001
Urban non-teaching	3050 (9.3%)	404,485 (18.5%)	
Urban teaching	29,040 (89.0%)	1,654,880 (75.8%)	
Bed size of the hospital			
Small	2930 (9.0%)	386,655 (17.7%)	<.001
Medium	7110 (21.8%)	621,069 (28.5%)	
Large	22,600 (69.2%)	1,174,060 (53.8%)	
Comorbidities			
Hyperlipidemia	17,440 (53.4%)	1,108,850 (50.8%)	<.001
Hypertension	24,825 (76.1%)	1,732,729 (79.4%)	<.001
Chronic heart failure	19,430 (59.5%)	1,200,865 (55.0%)	<.001
Prior MI	5370 (16.5%)	288,320 (13.2%)	<.001
Prior PCI	3960 (12.1%)	210,910 (9.7%)	<.001
Prior CABG	5740 (17.6%)	308,885 (14.2%)	<.001
Obesity	8060 (24.7%)	458,445 (21.0%)	<.001
Smoker/tobacco user	12,255 (37.5%)	864,660 (39.6%)	.001
COPD	5990 (18.4%)	542,430 (24.9%)	<.001
Obstructive sleep apnea	6065 (18.6%)	257,030 (11.8%)	<.001
Prior stroke	2400 (7.4%)	249,605 (11.4%)	<.001
Liver disease	2530 (7.8%)	240,265 (11.0%)	<.001
Diabetes mellitus	10,445 (32.0%)	815,555 (37.4%)	<.001
CKD/ESRD	9685 (29.7%)	786,615 (36.1%)	<.001
Hypothyroidism	4095 (12.5%)	276,645 (12.7%)	.751
Nutritional anemia	1615 (4.9%)	147,410 (6.8%)	<.001
DCM	3345 (10.2%)	123,165 (5.6%)	<.001
HCM	420 (1.3%)	20,050 (0.9%)	.002
COVID-19	115 (0.4%)	72,850 (3.3%)	<.001

*Abbreviations:* CABG, coronary artery bypass graft; CKD/ESRD, chronic kidney disease/end-stage renal disease; COPD, chronic obstructive pulmonary disease; DCM, dilated cardiomyopathy; HCUP, Healthcare Cost and Utilization Project; HCM, hypertrophic cardiomyopathy; MI, myocardial infarction; PCI, percutaneous coronary intervention.

**Table 2:** Multivariate Analysis of Outcomes in the Ablation Group while Adjusting for Comorbidities and Demographics

	Adjusted OR	95% CI	*P* Value
In-hospital mortality	0.28	0.24–0.32	<.001
ST-elevated MI	0.32	0.29–0.35	<.001
Acute heart failure	0.99	0.93–1.07	.941
Cardiogenic shock	1.10	1.01-1.20	.036
MCS	1.32	1.17–1.49	<.001
MACE (death/STEMI/CS)	0.44	0.41–0.48	<.001
Tamponade	4.84	3.94–5.94	<.001
Sepsis	0.26	0.22–0.30	<.001
LOS ≥7 days	0.96	0.91–1.01	.104

*Abbreviations:* CI, confidence interval; CS, cardiogenic shock; LOS, length of stay; MACE, major adverse cardiovascular event; MCS, mechanical circulatory support; MI, myocardial infarction; OR, odds ratio; STEMI, ST-elevation myocardial infarction. Reference: No ablation group.

**Table 3:** Outcomes and Results of Propensity Score Matching Analysis

	Before Propensity Score Matching		After Propensity Score Matching		*P* Value
	Ablation	No Ablation	Ablation	No Ablation	
N (total)	32,640 (1.5%)	2,181,784 (98.5%)	6334 (50.0%)	6334 (50.0%)	
In-hospital mortality	1055 (3.2%)	277,490 (12.7%)	201 (3.17%)	569 (8.98%)	<.001
ST-elevated MI	2230 (6.8%)	371,420 (17.0%)	432 (6.82%)	1193 (18.83%)	<.001
Acute heart failure	9165 (28.1%)	536,155 (24.6%)	1781 (28.12%)	1751 (27.64%)	.552
Cardiogenic shock	2960 (9.1%)	173,540 (8.0%)	579 (9.14%)	451 (7.12%)	<.001
MCS	1660 (5.1%)	76,680 (3.5%)	319 (5.04%)	235 (3.71%)	<.001
MACEs (death/STEMI/CS)	5160 (15.8%)	646,830 (29.6%)	1002 (15.82%)	1799 (28.40%)	<.001
Tamponade	585 (1.8%)	7785 (0.4%)	113 (1.78%)	27 (0.43%)	<.001
Sepsis	1100 (3.4%)	307,675 (14.1%)	214 (3.38%)	655 (10.34%)	<.001
LOS ≥ 7 days	13,365 (40.9%)	970,480 (44.5%)	2593 (40.94%)	2575 (40.65%)	.745

Outcome sheet before and after propensity score matching.

### Baseline characteristics

Patients undergoing ablation were more often male (72.0% vs. 65.2%) and White (76.0% vs. 70.8%), whereas the no-ablation group had more women (34.8% vs. 28.0%) and Black patients (17.5% vs. 12.1%) (all *P* < .001). The ablation cohort was younger, with 9.3% aged 18–44 years versus 6.3% in the non-ablation group (*P* < .001). These patients were more frequently treated in urban teaching (89.0% vs. 75.8%) and large hospitals (69.2% vs. 53.8%), while rural (1.7% vs. 5.6%) and urban non-teaching centers (9.3% vs. 18.5%) saw fewer ablation cases (all *P* < .001). Private insurance was more common among patients undergoing ablation (29.6% vs. 18.7%), while Medicare predominated in the no-ablation group (65.2% vs. 56.7%) (*P* < .001). Ablated patients generally had fewer comorbidities, with rates as follows: hypertension (76.1% vs. 79.4%), smoking (37.5% vs. 39.6%), COPD (18.4% vs. 24.9%), stroke (7.4% vs. 11.4%), liver disease (7.8% vs. 11.0%), diabetes (32.0% vs. 37.4%), CKD/ESRD (29.7% vs. 36.1%), anemia (4.9% vs. 6.8%), and COVID-19 (0.4% vs. 3.3%) (all *P* ≤ .001). Conversely, however, ablation patients more often had hyperlipidemia (53.4% vs. 50.8%), obesity (24.7% vs. 21.0%), OSA (18.6% vs. 11.8%), DCM (10.2% vs. 5.6%), and heart failure (59.5% vs. 55.0%). Although etiology-specific VT coding was unavailable, the ablation group had higher rates of prior MI (16.5% vs. 13.2%), PCI (12.1% vs. 9.7%), CABG (17.6% vs. 14.2%), and HCM (1.3% vs. 0.9%) (all *P* < .01), suggesting a greater structural heart disease burden compared to the non-ablation group. Hypothyroidism was similar (12.5% vs. 12.7%; *P* = .751).

### In-hospital outcomes (unadjusted analysis)

**Figure 2:**
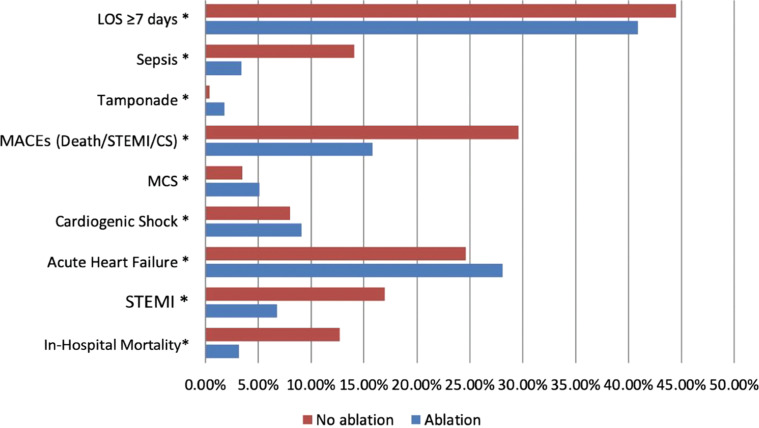
Unadjusted outcome rates stratified by ablation status. *Abbreviations:* CS, cardiogenic shock; LOS, length of stay; MACE, major adverse cardiac event; MCS, mechanical circulatory support; MI, myocardial infarction; STEMI, ST-elevation myocardial infarction.

Patients treated with catheter ablation experienced significantly lower in-hospital mortality (3.2% vs. 12.7%; *P* < .001). They also had a lower incidence of STEMI (6.8% vs. 17.0%; *P* < .001) and sepsis (3.4% vs. 14.1%; *P* < .001). The incidence of MACEs, defined as a composite of in-hospital mortality, STEMI, or cardiogenic shock, was significantly lower in the ablation group (15.8% vs. 29.6%; *P* < .001). Conversely, procedural complications such as cardiac tamponade (1.8% vs. 0.4%; *P* < .001), use of MCS (5.1% vs. 3.5%; *P* < .001), and cardiogenic shock (9.1% vs. 8.0%; *P* = .001) were significantly more frequent among ablated patients. Acute heart failure (28.1% vs. 24.6%; *P* < .001) was more common in the ablation group, while prolonged hospitalization (≥7 days) was less common in the ablation group (40.9% vs. 44.5%; *P* < .001) **(Figure 2)**.

### Adjusted outcomes (multivariable logistic regression)

**Figure 3:**
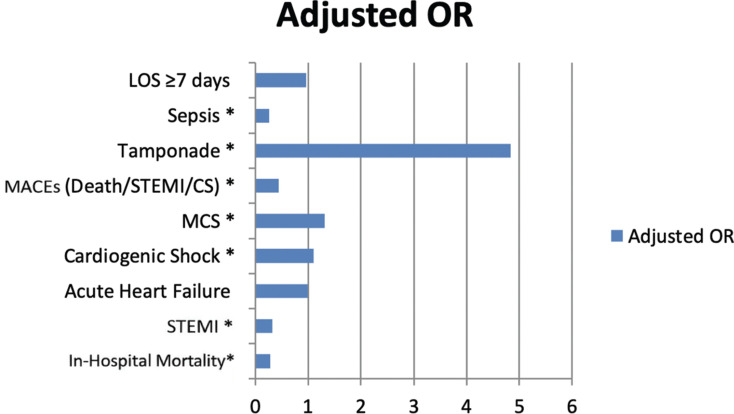
Adjusted odds ratios for adverse in-hospital outcomes. *Abbreviations:* CS, cardiogenic shock; LOS, length of stay; MACE, major adverse cardiac event; MCS, mechanical circulatory support; MI, myocardial infarction; OR, odds ratio; STEMI, ST-elevation myocardial infarction.

Following multivariate adjustment for demographic and clinical variables, catheter ablation remained independently associated with lower odds of in-hospital mortality (aOR, 0.28; 95% CI, 0.24–0.32; *P* < .001), STEMI (aOR, 0.32; 95% CI, 0.29–0.35; *P* < .001), and sepsis (aOR, 0.26; 95% CI, 0.22–0.30; *P* < .001). The composite MACE outcome also showed a reduced OR for ablation (aOR, 0.44; 95% CI, 0.41–0.48; *P* < .001). However, ablation was also associated with higher odds of cardiogenic shock (aOR, 1.10; 95% CI, 1.01–1.20; *P* = .036), a need for MCS (aOR, 1.32; 95% CI, 1.17–1.49; *P* < .001), and cardiac tamponade (aOR, 4.84; 95% CI, 3.94–5.94; *P* < .001). Acute heart failure (aOR, 0.99; 95% CI, 0.93–1.07; *P* = .941) and prolonged hospitalization (aOR, 0.96; 95% CI, 0.91–1.01; *P* = .104) showed no significant association with ablation after adjustment **(Figure 3)**.

### Propensity score–matched analysis

**Figure 4:**
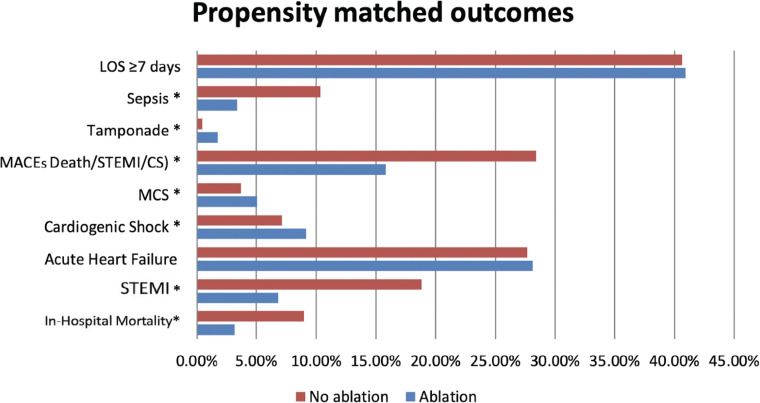
Propensity score–matched outcomes by ablation status. *Abbreviations:* CS, cardiogenic shock; LOS, length of stay; MACE, major adverse cardiac event; MCS, mechanical circulatory support; MI, myocardial infarction; STEMI, ST-elevation myocardial infarction.

A 1:1 propensity score–matched analysis using nearest-neighbor matching included 12,668 patients (6334 per group). In this matched cohort, catheter ablation was associated with a statistically significant reduction in in-hospital mortality (3.17% vs. 8.98%; *P* < .001), STEMI (6.82% vs. 18.83%; *P* < .001), and sepsis (3.38% vs. 10.34%; *P* < .001). The incidence of MACEs (death/STEMI/cardiogenic shock) was also lower in the ablation cohort (15.82% vs. 28.40%; *P* < .001). However, the ablation group had a higher association with cardiac tamponade (1.78% vs. 0.43%; *P* < .001), cardiogenic shock (9.14% vs. 7.12%; *P* < .001), and MCS (5.04% vs. 3.71%; *P* < .001). Acute heart failure (28.12% vs. 27.64%; *P* = .552) and prolonged hospital stay (≥7 days) (40.94% vs. 40.65%; *P* = .745) did not show statistically significant differences in the propensity score–matched analysis **(Figure 4)**.

## Discussion

Catheter ablation was associated with significantly improved short-term in-hospital outcomes compared with medical management alone. After multivariable adjustment and PSM, patients who underwent VT ablation experienced substantially lower in-hospital mortality, as well as lower rates of STEMI, sepsis, and the composite outcome of MACEs.

Catheter ablation, used in only 1.5% of VT patients without ICDs, was associated with lower rates of in-hospital death, STEMI, sepsis, and MACEs. After propensity matching, mortality fell from 8.98% to 3.17% and STEMI fell from 18.83% to 6.82% (*P* < .001). Tamponade and the need for mechanical support increased; heart failure rates and LOS were similar. Despite support from the 2022 European Society of Cardiology guidelines (class I/IIa after anti-arrhythmic failure; class IIb as first-line in select incessant VT) and a shift in nonichemic cardiomyopathy toward earlier use of drugs and ablation,^1,6^ use remains low: we report 1.5% versus NIS rates of 2.6% (2016–2019) and 3.1% (2018–2021).^7,8^ Most centers perform ≤10 VT ablations annually.^9^ Initial therapy often includes β-blockers, amiodarone, and mexiletine, with ablation performed for recurrent VT.^10,11^ High-resolution mapping systems like EnSite™ X (Abbott, Chicago, IL, USA) and Rhythmia (Boston Scientific, Marlborough, MA, USA) increasingly supplement or replace CARTO^®^ (JNJ MedTech, New Brunswick, NJ, USA), improving spatial resolution and efficiency.^10,11^ Utilization is uneven: ablated patients were typically younger, male, and White; women and Black patients were 14%–20% less likely to receive ablation,^10^ and rural residents were 35% less likely.^11^ These patterns accord with our findings. Yousuf et al. reported that privately insured patients were nearly twice as likely to undergo ablation,^12^ whereas Tang et al. found no robust income association, indicating narrowing yet persistent payer disparities.^10,12^ Institutional capacity amplifies inequity: Katz et al. observed fewer complications at high-volume centers (>25 VT ablations per year).^13^ In our data, about 90% of ablations occurred at large urban teaching hospitals; non-teaching and rural hospitals, despite about one-third of VT admissions, rarely offered ablation, consistent with the postcode lottery noted by Morcos et al.^11^ Advances in VT ablation technology, including the increasing adoption of high-density mapping systems, reflect broader temporal and center-level evolution in procedural practice. However, mapping platform use was not captured in the NIS and was not evaluated in this analysis; therefore, no inference can be made regarding the impact of specific mapping technologies on outcomes in the present study. Ablation was associated with lower in-hospital mortality (3.2% vs. 12.7%; aOR, 0.28). US databases concur (3.0% with ablation vs. 11%–13% without^14^), with similar ~70% reductions in structural heart disease cohorts.^15^ Trials align: VANISH halved death, VT storm, or ICD shocks at 6 months^3^; SMASH-VT (“Substrate Mapping and Ablation in Sinus Rhythm to Halt Ventricular Tachycardia”) reduced ICD therapies by 65%, with early mortality signals.^16^ Mechanistically, ablation terminates re-entry and attenuates sympathetic surges; a single ICD shock can triple sympathetic activity^17^ and may improve output and reduce inflammation and stretch or calcium abnormalities.^18^ Nevertheless, 30-day mortality remains 4%–5%, concentrated in advanced disease, shock, or persistent inducible VT; failure to achieve noninducibility accounted for 44% of deaths. Long-term mortality approximates 40% overall, exceeding 50% in severe structural disease.^19^ Catheter ablation was associated with a 68% relative reduction in STEMI among VT admissions (aOR, 0.32). Population-level evidence on MI as an ablation outcome is limited, as registries often omit or bundle MI under general complications. When reported, periprocedural MI is rare (0.09% in a 120,000-patient Japanese cohort; under 0.2% at US centers),^20^ aligning with our ablated group rate of 1.8 per 1000 versus 5.5 per 1000 with medical therapy.^20–22^ Epicardial ablation–related coronary events are exceptionally uncommon, with 61 cases over 30 years.^20–22^ Mechanistically, sustained VT increases oxygen demand and shortens diastole; doubling the heart rate can provoke subendocardial ischemia even in normal hearts.^23^ Guidelines recognize tachyarrhythmia-induced supply–demand mismatch as a cause of type 2 MI.^24^ Clusters of ICD shocks raise troponin, indicating myocyte injury from catecholamine surges and mechanical stunning. By eliminating reentry, ablation slows the ventricular rate, reduces sympathetic drive and shocks, improves perfusion, and lowers demand-related infarction risk. Acute heart failure occurred in about 28% of ablated cases and 25%–28% of VT hospitalizations overall, with neutral odds between groups (aOR, 0.99). VANISH showed no significant difference in heart failure at 30 days (5.1% vs. 6.2%) or at 6 months.^25^ The International VT Ablation Center Collaboration likewise found no excess early heart failure; risk tracked with disease severity, notably New York Heart Association (NYHA) class IV, which predicted decompensation and 1-year mortality.^25^ In high-risk settings, worsening heart failure reflected baseline status (NYHA III or IV and prior failed ablation) rather than the procedure itself.^26^ The German Ablation Registry, Pulmonary disease, Age, Ischemic cardiomyopathy, NYHA class, Ejection fraction, Storm, Diabetes (PAINESD), and Santangeli et al. similarly linked post-ablation heart failure to substrate, storm severity, and comorbidities, rather than to procedural harm.^27^ Pathophysiology integrates chronic tachycardia-mediated calcium handling defects, neurohormonal activation, and myocyte loss, as well as catecholamine-driven afterload and contractility impairment during storms.^28^ Procedure-related factors (anesthesia, fluid and contrast load, rapid pacing, radiofrequency [RF] microvascular injury) can transiently raise filling pressures,^27^ explaining neutral short-term outcomes despite longer-term improvement with VT suppression. Cardiogenic shock occurred in about 9.1% of patients after ablation, slightly higher than with medical therapy. MCS was used in about 5% of patients (aOR, 1.32), consistent with registry reports of acute hemodynamic decompensation in 8%–12% and support use in 5%–10% of high-risk ablations.^29,30^ PAINESD score–based analyses show that scores of ≥15 points carry sharply higher risks of shock and MCS; in such patients, preemptive left ventricular (LV) support nearly halves periprocedural decompensation.^31,32^ By contrast, starting MCS after shock onset triples to quadruples mortality, indicating illness severity rather than procedural harm. Our neutral to slightly higher shock rates likely reflect high-risk referral bias. Sustained VT stresses weakened myocardium, and anesthesia, rapid pacing, and RF injury add further strain. Temporary MCS unloads the ventricle, maintains perfusion, and permits complete mapping; observational series report 70%–80% successful device weaning in refractory shock.^33,34^ MCS complications remain substantial, including bleeding, hemolysis, stroke, and limb ischemia in 10%–20%.^35^ Cardiac tamponade occurred in 1.8% of VT ablation admissions versus 0.4% with medical therapy (aOR, 4.84), matching rates in contemporary endocardial VT registries (1.0%–1.6%).^36,37^ Higher rates (3%–9%) accompany epicardial access, especially in nonischemic substrates or near the LV summit.^36–38^ Administrative datasets of mixed ablations report a lower baseline risk of around 0.3%.^39^ Preventive strategies include ultrasound or micro-puncture–guided epicardial access, intracardiac echocardiography for early effusion detection, cautious contact force and power over thin myocardium, and a pigtail drain for 12–24 h after extensive epicardial ablation.^37,38,40^ Sepsis is a well-recognized trigger for ventricular arrhythmias through cytokine-mediated electrophysiologic disturbances, metabolic derangements, and heightened sympathetic activation and is also a strong marker of critical illness and poor prognosis. Patients presenting with active infection or septic physiology are less likely to be referred for invasive electrophysiologic procedures, introducing substantial selection bias. Accordingly, the higher sepsis rates in the non-ablation cohort likely reflect greater baseline illness severity, metabolic instability, and competing clinical priorities, rather than harm avoidance attributable to ablation. Sepsis occurred in 0.6% after ablation, a 74% lower rate than with medical therapy (aOR, 0.26), consistent with infection rates under 1% despite overall complication rates of 5%–9% in procedural registries.^41,42^ In broader critical care cohorts, sepsis affects 4%–8% of arrhythmia admissions, and pre-existing infection predicts VT storm and mortality.^43,44^ Cytokine-mediated potassium current suppression, nitric oxide–related calcium disturbance, and catecholamine-driven action potential shortening likely promote arrhythmogenesis.^44^ With a lack of timing, severity, source control, or vasopressor requirements, causal inference is limited. Thus, reduced sepsis rates in the ablation group should be interpreted as reflecting patient selection. Ablation may mitigate this by removing the VT focus, reducing sympathetic discharge and shocks, and limiting invasive exposure; antibiotic prophylaxis and ultrasound-guided access keep bacteremia below 0.3%.^45^ LOS did not differ after adjustment (aOR, 0.96). Prior reports vary, with longer stays in elective series (median, 6 vs. 4 days) and shorter stays in recent single-center cohorts (median, 3 days); prolonged stay tracks with NYHA class, RV dysfunction, and urgent presentation rather than procedural complexity.^46^ Emergent ablation registries report that 44% of unstable patients required hospital stays longer than 7 days or died early, with outcomes driven by disease severity rather than the procedure.^26^ Our findings likely reflect a balance between monitoring needs and the benefits of rhythm control, yielding comparable LOS times. Ablation may shorten hospitalization by reducing recurrent VT, ICD shocks, and catecholamine surges—factors linked to fewer readmissions and lower resource use in other cohorts.^47^ Countervailing procedural factors include vasodilation from general anesthesia—requiring vasopressors, fluids, and contrast load that may worsen congestion—the need for extended monitoring while sheaths remain, and complications such as effusion or bleeding that prolong stays (especially in high-risk patients), sometimes increasing the length of stay fourfold. Streamlined protocols, ultrasound-guided access, early sheath removal, and early mobilization may reduce LOS. At present, underlying disease severity remains the principal driver of admissions exceeding 7 days.^48^ An important consideration in interpreting our findings is the higher prevalence of ischemic heart disease markers, including prior myocardial infarction, PCI, and CABG, among patients who underwent VT ablation. Because the NIS does not reliably distinguish ischemic from nonischemic VT, these findings likely reflect referral and treatment patterns rather than causal effects of the substrate type. Patients with ischemic cardiomyopathy and scar-related VT are more commonly referred to tertiary centers with advanced electrophysiology capabilities, where catheter ablation is more readily available, whereas patients with nonischemic cardiomyopathy are less frequently offered ablation in non-specialized or lower-volume hospitals. Consequently, patients with nonischemic or idiopathic VT may be under-represented in the ablation cohort. This substrate-related referral bias may influence observed outcome differences, limiting the generalizability of our findings across all VT etiologies.

### Strengths

A principal strength of this study is its national scope. Leveraging the HCUP NIS dataset, a stratified 20% sample of US community hospital discharges, we assembled a large, demographically and geographically diverse VT cohort, enhancing external validity. The 2016–2021 window reflects contemporary practice (contact force catheters, high-density mapping, guideline-directed therapy). With more than 30,000 ablations and more than two million non-ablated admissions, the study was powered to evaluate both common and infrequent outcomes. Confounding was addressed with multivariable adjustment and PSM, and findings were consistent across sex, race, and hospital-volume strata. Restricting to ICD-naive admissions enabled estimation of the direct association between ablation and acute in-hospital outcomes, independent of device therapy.

### Limitations

This study has several important limitations inherent to its retrospective, observational design using administrative data. ICD-10 coding may have resulted in misclassification of VT subtype, ablation procedures, and complications, and the NIS lacks granular clinical information such as VT burden, ischemic acuity, scar characteristics, LV function, inducibility, procedural success, and illness severity, leaving residual confounding despite extensive multivariable adjustment and PSM. Selection bias and confounding by indication are likely, as patients referred for ablation may differ systematically from those managed medically with respect to hemodynamic stability, frailty, and clinician judgment. Additionally, the NIS captures only index hospitalizations, precluding assessment of readmissions or long-term outcomes. Importantly, VT etiology cannot be reliably classified as ischemic, nonischemic, or idiopathic; thus, observed associations may partially reflect substrate-related referral patterns rather than treatment effects alone, and findings may not generalize equally to patients with nonischemic or idiopathic VT.

## Conclusion

In this large national cohort of VT patients without prior ICDs, catheter ablation was used in only a small fraction of cases. Still, it was associated with lower rates of in-hospital death, STEMI, and sepsis. As an observational study, these findings warrant prospective confirmation and may support earlier consideration of ablation in carefully selected ICD-naive patients; efforts to improve equitable access are needed.

## Note

The included supplementary tables present variables and coding for ICD-naive VT ablation (NIS 2016–2021). These supplementary tables together expand the coding table with roles, precise code positions, and analysis notes.

## References

[1] Al-Khatib SM, Stevenson WG, Ackerman MJ (2018). 2017 AHA/ACC/HRS guideline for management of patients with ventricular arrhythmias and the prevention of sudden cardiac death: executive summary. A report of the American College of Cardiology/American Heart Association Task Force on Clinical Practice Guidelines and the Heart Rhythm Society. J Am Coll Cardiol.

[2] Kuck KH, Schaumann A, Eckardt L, VTACH Study Group (2010). Catheter ablation of stable ventricular tachycardia before defibrillator implantation in patients with coronary heart disease (VTACH): a multicentre randomised controlled trial. Lancet.

[3] Sapp JL, Wells GA, Parkash R (2016). Ventricular tachycardia ablation versus escalation of antiarrhythmic drugs. N Engl J Med.

[4] Santangeli P, Tung R, Xue Y (2019). Outcomes of catheter ablation in arrhythmogenic right ventricular cardiomyopathy without background implantable cardioverter defibrillator therapy: A multicenter international ventricular tachycardia registry. JACC Clin Electrophysiol.

[5] Li L, Ding L, Wu L (2024). Efficacy of catheter ablation for ventricular tachycardia in ischemic cardiomyopathy patients without an ICD implantation. Heart Rhythm.

[6] Middelfart C, Tønnesen J, Zörner CR (2024). Two decades of SVT ablation in Denmark: a trend towards higher age, more comorbidity, and less prior use of antiarrhythmic and rate-limiting pharmacotherapy-a nationwide registry-based Danish study. J Interv Card Electrophysiol.

[7] Ferraro B, Thwe EE, Shah K, Modi V, Stevens S, Shirani J (2022). Abstract 12155: Trends in catheter ablation of ventricular tachycardia in patients with ischemic and nonischemic cardiomyopathy: a national inpatient sample trend in the years 2016-2019. Circulation.

[8] Sheffeh MA, Sheffeh J, Baqal O (2024). Abstract 4144666: Outcomes of ventricular tachycardia ablation among patients with chronic kidney disease: insights from the national inpatient sample database 2018-2021. Circulation.

[9] Könemann H, Ellermann C, Zeppenfeld K, Eckardt L (2023). Management of ventricular arrhythmias worldwide: comparison of the latest ESC, AHA/ACC/HRS, and CCS/CHRS guidelines. JACC Clin Electrophysiol.

[10] Tang AB, Akinrimisi OP, Ziaeian B (2024). Sex, race, and rural-urban disparities in ventricular tachycardia ablations. JACC Clin Electrophysiol.

[11] Morcos R, Malik S, Hanna P (2024). Ventricular tachycardia ablation across age groups: Outcomes, trends and demographics. Insights from the National Inpatient Sample Database. Heart Rhythm O2.

[12] Yousuf OK, Zusterzeel R, Sanders W (2018). Trends and outcomes of catheter ablation for ventricular tachycardia in a community cohort. JACC Clin Electrophysiol.

[13] Katz DF, Turakhia MP, Sauer WH (2015). Safety of ventricular tachycardia ablation in clinical practice: findings from 9699 hospital discharge records. Circ Arrhythm Electrophysiol.

[14] Raj K, Singh Dhaliwal J, Han FT (2024). Outcomes of ventricular tachycardia ablation with intracardiac echocardiography-a retrospective cohort study of the national inpatient sample. Eur Heart J.

[15] Tan JL, Jin C, Lee JZ, Gaughan J, Iwai S, Russo AM (2022). Outcomes of catheter ablation for ventricular tachycardia in patients with sarcoidosis: insights from the National Inpatient Sample database (2002-2018). J Cardiovasc Electrophysiol.

[16] Reddy VY, Reynolds MR, Neuzil P (2007). Prophylactic catheter ablation for the prevention of defibrillator therapy. N Engl J Med.

[17] Tsuji Y, Dobrev D (2018). Electrical storm: mechanistic and therapeutic considerations to avoid death in the survivors. J Thorac Dis.

[18] Tsuji Y, Dobrev D (2018). Prognostic impact of electrical storm in patients with implantable cardioverter defibrillators: mechanistic and therapeutic considerations to reduce the risk of death. Int J Cardiol.

[19] Rottner L, Metzner A, Hochadel M (2025). Ten-year outcomes and predictors of mortality following catheter ablation of ventricular tachycardia. J Am Heart Assoc.

[20] Pothineni NV, Kancharla K, Katoor AJ (2019). Coronary artery injury related to catheter ablation of cardiac arrhythmias: a systematic review. J Cardiovasc Electrophysiol.

[21] Waciński P, Głowniak A, Czekajska-Chehab E, Dąbrowski W, Wójcik J, Wysokiński A (2013). Acute left main coronary artery occlusion following inadvertent delivery of radiofrequency energy during ventricular tachycardia ablation successfully treated by rescue angioplasty with stenting: a two-year follow-up. Cardiol J.

[22] Yune S, Lee WJ, Hwang JW, Kim E, Ha JM, Kim JS (2014). Acute myocardial infarction after radiofrequency catheter ablation of typical atrial flutter. J Korean Med Sci.

[23] Hoffman JIE, Buckberg GD (2014). The myocardial oxygen supply: demand index revisited. J Am Heart Assoc.

[24] DeFilippis AP, Chapman AR, Mills NL (2019). Assessment and treatment of patients with type 2 myocardial infarction and acute nonischemic myocardial injury. Circulation.

[25] Tzou WS, Tung R, Frankel DS (2017). Ventricular tachycardia ablation in severe heart failure: an international ventricular tachycardia ablation center collaboration analysis. Circ Arrhythm Electrophysiol.

[26] Uetake S, Hasegawa K, Kurata M (2024). Emergent ablation for ventricular tachycardia: predictors of prolonged hospitalization and mortality. JACC Clin Electrophysiol.

[27] Santangeli P, Muser D, Zado ES (2015). Acute hemodynamic decompensation during catheter ablation of scar-related ventricular tachycardia: incidence, predictors, and impact on mortality. Circ Arrhythm Electrophysiol.

[28] Eifling M, Razavi M, Massumi A (2011). The evaluation and management of electrical storm. Tex Heart Inst J.

[29] Virk SA, Keren A, John RM, Santageli P, Eslick A, Kumar S (2019). Mechanical circulatory support during catheter ablation of ventricular tachycardia: indications and options. Heart Lung Circ.

[30] Kautzner J, Hašková J, Stojadinovič P, Peichl P, Wichterle D (2024). Percutaneous mechanical support in catheter ablation of ventricular arrhythmias: hype or hope?. Europace.

[31] Muser D, Liang JJ, Castro SA (2018). Outcomes with prophylactic use of percutaneous left ventricular assist devices in high-risk patients undergoing catheter ablation of scar-related ventricular tachycardia: a propensity-score matched analysis. Heart Rhythm.

[32] Muser D, Castro SA, Liang JJ, Santangeli P (2018). Identifying risk and management of acute haemodynamic decompensation during catheter ablation of ventricular tachycardia. Arrhythm Electrophysiol Rev.

[33] Reddy YM, Chinitz L, Mansour M (2014). Percutaneous left ventricular assist devices in ventricular tachycardia ablation: multicenter experience. Circ Arrhythm Electrophysiol.

[34] Ballout JA, Wazni OM, Tarakji KG (2020). Catheter ablation in patients with cardiogenic shock and refractory ventricular tachycardia. Circ Arrhythm Electrophysiol.

[35] Tavazzi G, Dammassa V, Colombo CNJ (2022). Mechanical circulatory support in ventricular arrhythmias. Front Cardiovasc Med.

[36] Sacher F, Roberts-Thomson K, Maury P (2010). Epicardial ventricular tachycardia ablation a multicenter safety study. J Am Coll Cardiol.

[37] Vemulapalli HS, Rodriguez-Riascos JF, Muthu P (2024). Epicardial access for ventricular tachycardia and premature ventricular complexes ablation: an institutional experience. Heart Rhythm O2.

[38] Della Bella P, Brugada J, Zeppenfeld K (2011). Epicardial ablation for ventricular tachycardia: a European multicenter study. Circ Arrhythm Electrophysiol.

[39] Li A, Buch E, Boyle NG, Shivkumar K, Bradfield JS (2018). Incidence and significance of adhesions encountered during epicardial mapping and ablation of ventricular tachycardia in patients with no history of prior cardiac surgery or pericarditis. Heart Rhythm.

[40] Xu X, Meng X, Ma F (2020). Delayed cardiac tamponade following catheter ablation of frequent premature ventricular complexes: a case report. BMC Cardiovasc Disord.

[41] Eckardt L, Doldi F, Anwar O (2023). Major in-hospital complications after catheter ablation of cardiac arrhythmias: individual case analysis of 43 031 procedures. Europace.

[42] Ding WY, Pearman CM, Bonnett L (2022). Complication rates following ventricular tachycardia ablation in ischaemic and non-ischaemic cardiomyopathies: a systematic review. J Interv Card Electrophysiol.

[43] Schwartz A, Brotfain E, Koyfman L, Klein M (2015). Cardiac arrhythmias in a septic ICU population: a review. J Crit Care Med (Targu Mures).

[44] Shahreyar M, Fahhoum R, Akinseye O, Bhandari S, Dang G, Khouzam RN (2018). Severe sepsis and cardiac arrhythmias. Ann Transl Med.

[45] Benali K, Khairy P, Hammache N (2023). Procedure-related complications of catheter ablation for atrial fibrillation. J Am Coll Cardiol.

[46] Gurin MI, Xia Y, Tarabanis C (2024). Catheter ablation compared to medical therapy for ventricular tachycardia in sarcoidosis: nationwide outcomes and hospital readmissions. Am Heart J Plus.

[47] Winterfield JR, Kent AR, Karst E (2018). Impact of ventricular tachycardia ablation on health care utilization. Heart Rhythm.

[48] Calcagno T, Cesmat A, Sipko J (2025). Prolonged hospitalization after catheter ablation of ventricular tachycardia: predictors and outcomes. Heart Rhythm.

